# Brown adipose tissue: endocrine determinants of function and therapeutic manipulation as a novel treatment strategy for obesity

**DOI:** 10.1186/s40608-014-0013-5

**Published:** 2014-08-22

**Authors:** Narendra L Reddy, Bee K Tan, Thomas M Barber, Harpal S Randeva

**Affiliations:** Clinical Sciences Research Laboratories, Division of Metabolic and Vascular Health, Warwick Medical School, University of Warwick, University Hospitals Coventry and Warwickshire, Clifford Bridge Road, Coventry, CV2 2DX UK; Warwickshire Institute for Study of Diabetes, Endocrinology and Metabolism, University Hospitals Coventry and Warwickshire NHS Trust, Clifford Bridge Road, Coventry, CV2 2DX UK; Obstetrics and Gynaecology, Birmingham Heartlands and Solihull Hospitals, Heart of England NHS Foundation Trust, Birmingham, B9 5SS UK

**Keywords:** Brown adipose tissue, Obesity, Hormone

## Abstract

**Introduction:**

Recent observation of brown adipose tissue (BAT) being functional in adult humans provides a rationale for its stimulation to increase energy expenditure through ‘adaptive thermogenesis’ for an anti-obesity strategy. Many endocrine dysfunctions are associated with changes in metabolic rate that over time may result in changes in body weight. It is likely that human BAT plays a role in such processes.

**Review:**

In this brief review article, we explore the endocrine determinants of BAT activity, and discuss how these insights may provide a basis for future developments of novel therapeutic strategies for obesity management.

A review of electronic and print data comprising original and review articles retrieved from PubMed search up to December 2013 was conducted (Search terms: brown adipose tissue, brown fat, obesity, hormone). In addition, relevant references from the articles were screened for papers containing original data.

**Conclusion:**

There is promising data to suggest that targeting endocrine hormones for BAT modulation can yield a cellular bioenergetics answer for successful prevention and management of human obesity. Further understanding of the physiological link between various endocrine hormones and BAT is necessary for the development of new therapeutic options.

**Electronic supplementary material:**

The online version of this article (doi:10.1186/s40608-014-0013-5) contains supplementary material, which is available to authorized users.

## Introduction

According to the World Health Organization (WHO) report, worldwide obesity rates have more than doubled since 1980. Global figures from 2008 showed that 1.5 billion adults were overweight and that obesity affected 200 million men and 300 million women, with the numbers expected to rise exponentially [1]. Obesity is associated with significant morbidity and mortality that result from the related complications of type 2 diabetes mellitus (T2DM), non-alcoholic fatty liver disease, cardiovascular events, obstructive sleep apnoea, musculoskeletal and psychiatric diseases, and various malignancies [2]. In 2010, overweight and obesity were estimated to cause 3.4 million deaths, 3.9% of years of life lost, and 3.8% of disability-adjusted life-years (DALYs) worldwide [3]. Obesity, in 1980’s was limited to affluent countries such as North America, Western Europe and Australasia, but now manifests as a true pandemic, with its increasing prevalence in developing countries such as India, China and Brazil, and spreading even to sub-Saharan Africa [4],[5], placing an enormous financial burden on the global economy.

The management of obesity through lifestyle is notoriously difficult and the resulting effects on weight are variable and often transient. Weight regain following weight loss is common and results from a number of mechanisms that redress any loss of energy storage capacity. Such mechanisms include changes in the levels of appetite-regulating hormones following weight loss that encourage weight recovery [6]. Weight loss also reduces energy expenditure [7] and brown adipose tissue (BAT) activity, and this combined with enhanced appetite promotes weight regain. Current therapeutic options for obesity management are limited following recent withdrawals of sibutramine and rimonabant amid safety concerns, and problems relating to the supply, unacceptable side-effect profile and long-term efficacy of orlistat [8]. Despite its effectiveness as a weight-loss intervention, bariatric surgery is only applicable to a sub-group of obese patients who meet funding criteria and as such, does not represent a practical solution to the global obesity epidemic [9]. Given the limitations of current therapies, the current global obesity epidemic and escalating incidence of obesity-related deaths, it is imperative to identify novel and effective therapeutic options for obesity.

Obesity results when energy intake exceeds expenditure chronically. Therapeutic strategies for obesity have mainly targeted caloric restriction through central appetite suppression and inhibition of fat absorption [10]. Compared with those acting on central appetite regulation, therapies acting peripherally may prove beneficial whilst causing fewer harmful effects [11]. The body is, by default, genetically predisposed to store energy in preparation for prolonged periods of starvation [12]. Even minor weight-loss through appetite suppression is often redressed through multiple peripheral counter-regulatory mechanisms to maintain ‘isoenergetic’ conditions [6]. Centrally acting drugs can potentially cause adverse psychotropic side effects through cross-reactivity with a variety of other receptors within complex central circuits (such as the endocannabinoid receptor blocker, Rimonabant) [10]. The concept of increasing energy expenditure through therapeutic manipulation of *peripheral* mechanisms is therefore attractive and worthy of focused research and development.

The main physiological function of BAT, to generate heat for the organism to protect against development of hypothermia, has been well understood for nearly 50 years [13]. Recent studies using ^18^fluoro-labelled 2-deoxyglucose (FDG) positron emission tomography computed tomography (PET-CT) have demonstrated the presence of BAT depots in the axillary, paravertebral, supraclavicular and cervical regions in adult humans [14]-[16]. Data from various animal studies have demonstrated that through BAT activation, triglyceride stores within white adipose tissue (WAT) can be utilized for heat generation through modulation of adaptive thermogenesis [17]. Therapeutic manipulation of human BAT therefore represents a novel mechanism to promote weight-loss. It is noted that endocrine disorders such as phaeochromocytoma and thyrotoxicosis play a role in activating BAT [18],[19]. To maximize its future therapeutic potential, it is important to appreciate the mechanisms by which endocrine dysfunction influences human BAT activity. In this brief review article, we explore the main mechanisms linking various endocrine hormones and human energy expenditure, mediated by effects on BAT activity.

### BAT energetics

**Figure 1:**
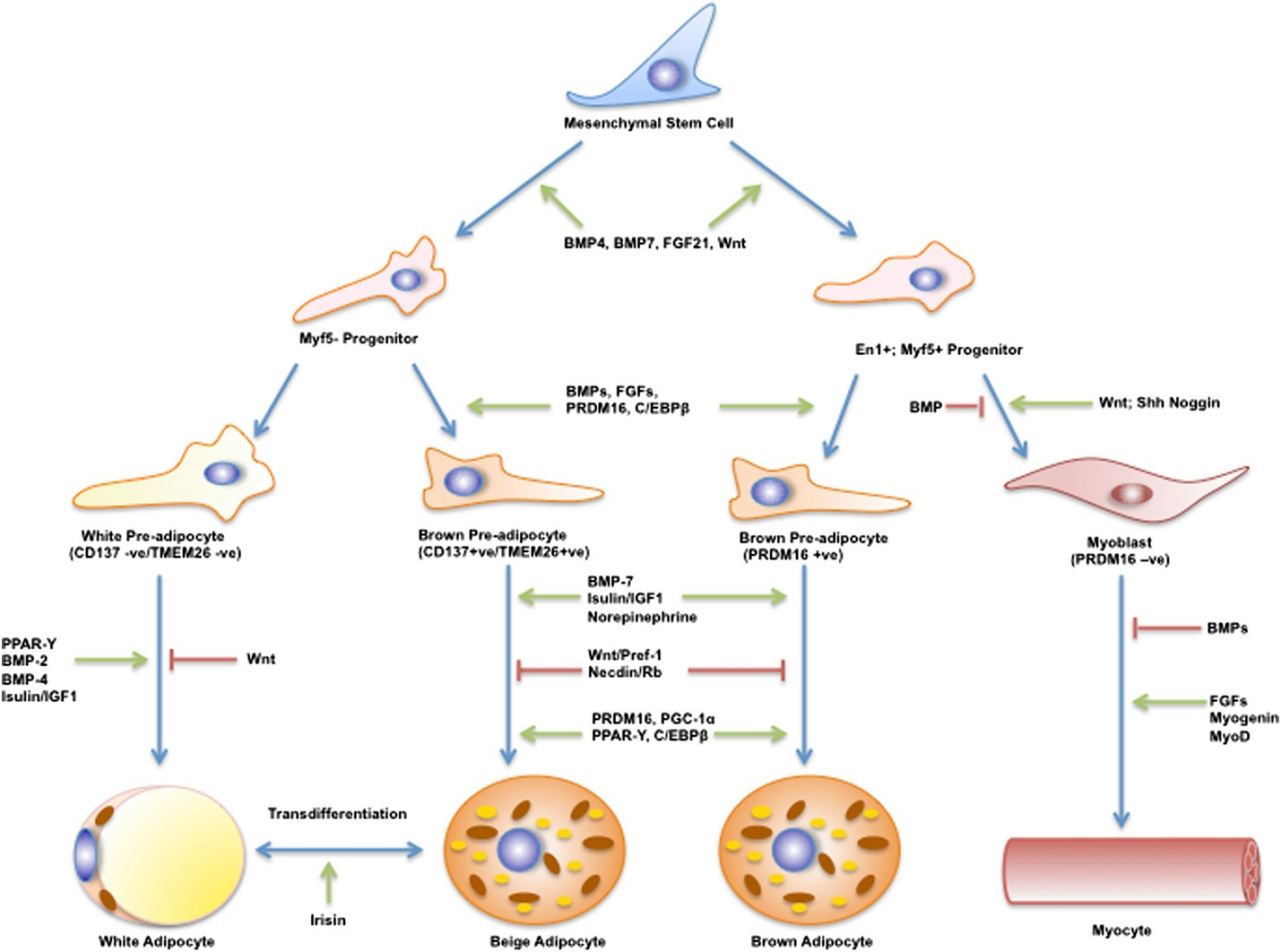
**Origin and transcriptional regulation of brown adipocyte.** Multipotent mesenchymal stem cells commit to brown adipocyte lineage following developmental triggers such as bone morphogenic proteins (BMP) and fibroblast growth factors (FGFs) leading on to cascade resulting in a fully developed brown adipocyte. Myf5-expressing progenitors give rise to skeletal muscle and brown adipocytes in traditional sites such as interscapular area. Myf5-negative progenitors are common precursors for both white adipocyte and recruitable brown adipocyte or beige adipocyte. Beige adipocyte is formed from either the trans differentiation from white adipose tissue in response to cues such as Irisin or from recruitable brown preadipocyte.

There are two main types of adipose tissue, white adipose tissue (WAT) and BAT that have evolved for completely different purposes: to survive famine and prevent hypothermia respectively. WAT and BAT, as energy storage and thermogenic tissues respectively, therefore evolved to protect mammalian organisms from important environmental threats, including lack of food and exposure to cold climates [20]. In addition to WAT and BAT, a third intermediate-type of adipose tissue that is termed ‘beige’ has recently been identified. Adipocytes from beige adipose tissue (BeAT) depots resemble white adipocytes but possess the classical properties of brown adipocytes. Partial success noted in animal models in converting WAT to BeAT, has set a tone in BAT research field to replicate the concept in humans too [21],[22]. The characteristic features of WAT, BAT and BeAT, and the origin of BAT are shown in Table 1 and Figure 1 respectively.

**Table 1:** **Morphological features of BAT, WAT and BeAT**

	WAT	BAT	BeAT
Cell shape	Variable, but classically spherical	Polygonal	Resembles WAT
Cell size	Variable, but large (25-200 μm)	Comparatively small (15-60 μm)	Variable
Nucleus	Peripheral, flattened	Central, round or oval in shape	To be determined
Cytoplasm	Thin, peripheral rim	Large volume evenly distributed throughout cell	To be determined
Lipid content	Single large droplet occupying up to 90% of cell volume	Multiple small lipid droplets	To be determined
Mitochondria	Few	Abundant	Intermediate
Endoplasmic reticulum (ER)	Little, but recognizable as rough and smooth ER	Present, but poorly developed	To be determined
Tissue organization	Small lobules of densely packed cells	Lobular, gland-like	To be determined
Cell content	Multiple other cell types present	Few other cell types present	Few other cell types present
Vascularity	Adequate	Highly vascularised	To be determined
Gene expression	PPAR-gamma, aP2, Adiponectin, adipsin, perilipin	UCP-1, PGC-1alpha, β-3 adreno receptor (ARB3), PRDM16, de-iodinase type II (D2)	Low UCP1, but activated by cAMP stimulation
Cell markers	CD34, ABCG2, ALDH	EVA1, EBF3, FBXO31	CD137, TMEM26, TBX1

Heat production plus external work account for the average daily metabolic rate or total energy expenditure (TEE). TEE can be classically divided into resting metabolic rate (RMR; normally 55–65% of TEE), activity related energy expenditure (AEE; normally 25–35% of TEE), and diet-induced thermogenesis (DIT) (about 10% of TEE) [23],[24]. Alternative classification is obligatory energy expenditure, which includes RMR, involuntary AEE and obligatory part of DIT, and facultative thermogenesis, which includes voluntary AEE, cold-induced non-shivering thermogenesis (NST), cold-induced shivering thermogenesis, and facultative part of DIT [23].

Cold-induced activation of BAT has resulted in a high incidence (60% to 96%) of detection as shown in recent PET studies [25],[26]. The presence of the 32 kDa uncoupling protein-1 (UCP1) in BAT mitochondria enables heat dissipation rather than generation of adenosine tri-phosphate (ATP) [27], thereby resulting in non-shivering thermogenesis (NST). Although controversial, BAT is thought to influence DIT through sympathetic nervous system activity via UCP1 [27],[28]. Using PET studies with radio-labeled fatty acid tracers, Ouellet *et al.* quantified BAT oxidative metabolism, glucose and non-esterified fatty acid (NEFA) turnover in 6 healthy human subjects, demonstrating unequivocally that BAT contributes to energy expenditure in humans [29]. Extrapolating rodent experiments of thermogenic potential of BAT (300 W/kg), Rothwell and Stock calculated that 40-50 g of BAT in humans, might account for 20% of total energy expenditure [30]. Human PET studies estimated that maximal activation of 63 g of BAT would result in 4.1 kg of weight loss during one year [14]. Two independent but congruent human studies estimated an energy expenditure of 200–400 kcal/day, a 10 to 20% rise in daily basal metabolic rate through BAT activation [31],[32]. Therefore, the glucose disposal [33] and triglyceride clearance properties of BAT [34], when fully utilized may act as an energy sink. There are three ways in which enhanced energy expenditure through manipulation of BAT could be theoretically achieved: i) maximal and continual activation of BAT; ii) trans-differentiation of WAT to BAT (to form BeAT), and; iii) transplantation of BAT stem cells.

The presence of BAT in adult humans represents a potentially important therapeutic target for future novel weight-loss strategies. The origins and functions of BAT, WAT and BeAT differ in important ways, and studies on the energetics of BAT have shown promising results. In the next sections, we discuss the main endocrine determinants of human BAT activity, and how each of these mechanisms could be therapeutically manipulated for promotion of weight-loss.

## Review of endocrine determinants of BAT activity

### Thyroid and BAT

We have known for over a century that thyroid hormone (TH) increases metabolic rate and thermogenesis in homeothermic species, and hence is an important physiological modulator of energy homeostasis [35],[36] TH stimulates both obligatory and facultative thermogenesis [37] and plays an important role in the regulation of lipid metabolism within adipose tissue [38],[39]. TH also enhances oxidative phosphorylation through induction of mitochondrial biogenesis and modulation of the expression of genes implicated in the regulation of the mitochondrial respiratory chain [40]. The weight gain and decreased cold tolerance observed in individuals with hypothyroidism, and the weight loss and sweating/heat intolerance observed in patients with hyperthyroidism, are predictable clinical manifestations of alterations in BAT activity [41]. It follows therefore that differences in BAT quantity and/or activity between individuals may also influence the clinical manifestations of hypo- or hyperthyroid states. This may also explain the inter-individual variability of weight changes and heterogeneity of other clinical manifestations of dysthyroid states.

The physiological effects of TH are exerted at the level of transcription through the thyroid receptors (TR): TRα and TRβ [42]. TRβ mediates thyronine (T3) induced *UCP1* gene expression, whilst the TRα isoform through T3 regulates facultative thermogenesis in BAT [43]. Type 2 deiodinase (D2) plays an essential role in mediating the full thermogenic response of BAT to adrenergic stimulation via increased thyroxine (T4) to T3 conversion within this tissue [44]. From a therapeutic perspective, it would be desirable to selectively activate TRβ for UCP1 stimulation to avoid the widespread unwanted effects of TRα, the predominant receptor in non-BAT tissues. Thyroid hormone analogues have been explored with variable outcomes. GC-1 compound, a selective TRβ agonist, induces *UCP1* gene expression in rats [43], improves glucose homeostasis [45], increases energy expenditure and reduces fat mass and plasma cholesterol [46]. High-fat feeding and concurrent treatment with the TRβ-selective agonist GC-24 (with a 40-fold higher affinity for TRβ than TRα) resulted in only a partial improvement in metabolic control, probably related to acceleration of resting metabolic rate [47]. Treatment with another TRβ-selective agonist, KB-41 in rats resulted in a 6-8% weight-loss with significant improvements in glucose homeostasis, cholesterol and triglyceride levels without affecting heart rate, probably due to lack of TRα effects [45].

There are also some promising data from human studies that implicate thyroid hormones having important effects on BAT activity. T3 treatment of differentiated human multipotent adipose-derived stem cells *in vitro* induces UCP1 expression and mitochondrial biogenesis through effects on TRβ [48]. Following thyroidectomy and subsequent treatment with thyroxine replacement therapy in a patient with papillary carcinoma, BAT activity was enhanced with concurrent weight-loss and remission of T2DM [49]. Thyroxine may cause ‘brownification’ of WAT [48], and holds immense potential given the mechanism of action in BAT, and hence needs to be robustly tested in humans.

### Catecholamines and BAT

Epinephrine causes vasodilatation and enhances glucose and oxygen consumption in skeletal muscle [50] whilst also enhancing thermogenesis in humans [51]. BAT is also activated in patients with phaeochromocytoma, (excess catecholamine producing benign adrenal medullary tumour) with increased UCP1 expression similar to levels in cold-exposed rodents [18],[52]. BAT activity is greater in patients with phaeochromocytoma [53],[54] due to over-activity of the sympathetic nervous system and elevated levels of circulating catecholamines, that in turn stimulate β3 adrenergic receptors, thereby activating *UCP1* expression via cyclic adenosine monophosphate (cAMP) and protein kinase-A (PKA) pathways [55]. Hadi *et al*. demonstrated active BAT to be present in 27% (26/96) of phaeochromocytoma patients undergoing FDG PET-CT scans [56], indicating higher detection rates compared to 5.37% (106/1972) of all cause PET-CT studies reported by Cypess and colleagues [16]. Recent human observational studies demonstrate a correlation between plasma metanephrine levels and BAT activity [57].

Nor-epinephrine action on β3-adrenergic receptor in mature human brown adipocyte is the most studied pathways. β3-adrenergic receptor would appear to be a convenient therapeutic target based on evidence from rodent studies using “selective” β3-agonists (CL-316,243) [58] and knockout mouse models [59]. β3-agonists have not yielded desirable results in humans due to differences in β3-receptor binding properties in humans and rodents. Second-generation β3-agonist trials in humans were unsuccessful due to poor oral bioavailability and unfavorable pharmacokinetics [60]. Another β3-agonist, L-796568, showed an initial increase in energy expenditure effect in 12 healthy obese subjects that failed to be sustained beyond 28 days [61],[62]. Catecholamines may also ‘brownify’ WAT. Two case reports of extensive brown fat deposits in omental and mesenteric regions detected on human FDG-PET scans indicate a possible role for catecholamines in the ‘browning’ of WAT [63],[64]. Therapeutically, catecholamine-like molecules may trans-differentiate WAT into BeAT, but such an approach would need to avoid the associated sympathomimetic effects to be safe.

### Glucocorticoids and BAT

Both BAT and WAT contain glucocorticoid receptors [65]. Excessive levels of glucocorticoids increase WAT mass and result in weight gain [66]. Conversely, glucocorticoids have an *inhibitory* effect on BAT activity in rodent models [67]. Glucocorticoids enhance appetite, stimulate lipolysis, suppress thermogenesis [68] (specifically *facultative* thermogenesis [69]) and profoundly suppress norepinephrine-induced *UCP1* activation [67]. Glucocorticoids also inhibit the expression and function of β1 and β3 adrenergic receptors within BAT. [70],[71] Corticosterone reduces NST and increases lipid storage within BAT in an *in vivo* rodent study, possibly as a result of steroid-induced hyperinsulinaemia [69]. Within rodent models, it has been observed that adrenalectomy results in stimulation of BAT thermogenesis and also weight-loss [72]. This mechanism is probably mediated through removal of glucocorticoid-induced hypothalamic inhibitory influences on BAT activity, and is reversed following glucocorticoid administration [72],[73]. A similar reduction in body fat mass was seen in a 46-year old female with Cushing’s syndrome following adrenalectomy [74]. The therapeutic challenge here would be to develop the beneficial effects of steroid depletion on metabolism and adipose-regulation whilst avoiding its potentially life-threatening effects.

### Mineralocorticoid and BAT

Mineralocorticoid receptors in rodent BAT, were first demonstrated by Zennaro and colleagues [75]. Following aldosterone treatment of a T37i cell line derived from hibernoma in mice, there was increased expression of adipogenic genes such as *Lpl* (lipoprotein lipase), *PPAR-γ* (Peroxisome proliferator receptor activated-gamma) (*PPAR-γ)* and *aP2* (adipocyte-specific fatty acid binding protein) [75],[76]. Treatment with aldosterone also results in inhibition of *Ucp1* expression, favouring lipid storage rather than heat dissipation [77],[78]. Within WAT, aldosterone induces inflammation resulting in the release of pro-inflammatory cytokines such as Interleukin-6 (IL-6), tumour necrosis factor-alpha (TNF-α) and Monocyte chemo attractant protein (MCP-1) [79]. Aldosterone also appears to inhibit thermogenesis within BAT, and also inhibits the differentiation of WAT into BAT [80]. Given that mineralocorticoids have a negative effect on BAT, it follows that aldosterone antagonists may represent a combined therapy for both hypertension and obesity (through possible activation of BAT). This also supports the findings that high aldosterone levels are noted in obesity-induced hypertension in humans, which reverses on weight loss [81].

### Growth hormone/Insulin Growth Factor-1 and BAT

BAT-status in growth hormone (GH)-deficient patients and acromegalics remains unknown. GH replacement in GH-deficient humans results in sustained improvement of body composition and reduction of insulin resistance [82],[83]. Conversely, GH excess in acromegalics promotes insulin resistance [82], resulting in dysglycaemia and hyperlipidaemia. GH replacement (1 mg/kg/day) for 10 days in experimental mice resulted in significant reduction of WAT mass, increased skeletal weight and reduction of insulin resistance. Despite an increase in *Ucp-1* mRNA by 2.8 fold, there was no change in the inter-scapular brown fat mass [84], although a substantial increase (2 to 6 fold) in inter-scapular brown fat mass was noted at higher doses of GH (3.5 mg/kg/day).

Insulin Growth Factor-1 (IGF-1) receptors are highly expressed in the plasma membrane of rat brown adipocytes [85]. *In vitro* studies in murine foetal brown adipocytes have shown that IGF-1 is intensely mitogenic and prevents TNF-α induced apoptosis [86],[87]. IGF-1 induces the expression of *Ucp-1* and CCAAT/enhancer binding protein alpha (C/EBP-α) in rat brown adipocyte primary-cell cultures [88]. Transient up-regulation of *Igf-1* gene expression and BAT hyperplasia was noted in rats exposed to cold (4°C) in the first 48 hours [89]. One of the factors influencing the dramatic rise in human foetal UCP-1 content during late gestation, especially prior to birth, is thought to be due to increased IGF-1 and IGF-2 levels [90]. There may therefore be a role for IGF-1 in BAT differentiation and activation, although the precise molecular mechanisms remain unclear. As a therapeutic strategy, the effect of GH or recombinant human IGF-1 (or truncated IGF-1) on BAT and WAT functioning is worth exploring.

### Prolactin and BAT

Functional prolactin receptors (PRLR) are highly expressed in both WAT and BAT and are essential for adipogenesis and adaptive thermogenesis [91]. Prolactin plays important roles in carbohydrate metabolism through its effects on pancreatic β-cell mass and energy homeostasis through lipid metabolism [92]. Prolactin suppression, through use of dopamine agonists in hyperprolactinaemic patients, results in metabolic effects [93]. Lactation in experimental mice is strongly and negatively associated with expression of thermogenic genes in BAT [94]. PRLR−/− male mice subjected to a high fat diet for 16 weeks exhibited resistance to weight-gain and a reduction in WAT compared to wild-type mice. These mice also showed 2–3 fold increased expression of BAT-specific markers (PR domain containing 16 [PRDM16], UCP1, PPAR-coactivator 1-alpha [PGC1α]) and brown-like adipocyte foci, indicating a possible role in BeAT differentiation from WAT [95]. Further studies are required to establish whether prolactin blockade by either dopamine agonists or pure prolactin receptor antagonists may represent a targeted approach for browning of human WAT.

### Sex hormones and BAT

Androgen and oestrogen receptors (ERα) are expressed in BAT in both sexes [96]. Furthermore, sex hormones play an important role in the BAT thermogenic program by acting at several steps of the lipolytic signal cascade and on UCP1 transcription control. Observations such as cessation of ovarian function at menopause resulting in weight-gain, loss of insulin sensitivity and increased incidence of cardiovascular disease [97], coupled with greater BAT activity in young females in PET-CT studies [16], fuel the argument that ovarian hormones probably influence BAT function. Ovariectomy in female rodents reduced BAT mitochondrial functionality through reduction in the oxidative capacity and anti-oxidant defenses. Furthermore, 17-β oestradiol (E2) supplementation partially reversed these effects indicating oestrogen’s partial influence on BAT [98]. There may also be non-oestrogenic ovarian signals stimulating BAT activity [98]. Interestingly, i*n vitro* cell culture studies by Rodriguez-Cuenca show a dual effect of 17-β oestradiol on the mitochondrial biogenic program [99],[100].

Addition of testosterone reduced norepinephrine-induced *Ucp1* mRNA expression in a dose-dependent manner in cultured rodent brown adipocytes, and these effects were reversed by flutamide (an androgen receptor antagonist) [101]. Furthermore, testosterone reduces the thermogenic and lipolytic capacity of BAT [100]. In contrast, progesterone is shown to have the opposite effect to that of testosterone on brown adipocytes [101] by positively stimulating mitochondriogenesis and BAT differentiation as demonstrated by an increase in the mRNA expression of the *GABPA-TFAM* axis and *PPAR-γ*, respectively [99]. These apparent opposite influences of testosterone and progesterone on BAT activity may explain the gender dimorphism displayed by BAT in human PET studies [16],[102]. Dehydroepiandrosterone (DHEA, a precursor sex steroid), when administered to obese and lean rats caused reduced food intake and enhanced energy expenditure resulting in weight-loss through increased expression of *Pgc-1α, Ucp1* and *β3-Ar*[103].

In summary, these animal studies demonstrate variable effects of sex hormones on BAT activity: testosterone appears to have a negative influence, oestrogen probably has a dual effect and progesterone and DHEA both appear to have positive influences on BAT activity. However, the increase in both, BAT amount and BAT activity in both sexes in human adolescents, (during peak surge of sex hormones) [104] fuels speculation that sex hormones may have a strong influence on BAT. Therefore it is worth exploring the influences of flutamide, selective oestrogen-receptor modulators (SERMs) and DHEA on human BAT activity.

### Insulin and BAT

In cultured murine brown and white adipose tissue, insulin has a role in differentiation of pre-adipocytes into adipocytes [105]. Furthermore, insulin-signaling in BAT is similar to that of WAT and other tissues, displaying similar anabolic effects of glucose uptake and lipid accretion [106]. The studies suggest that uptake of glucose into BAT is both insulin-mediated (mainly occurring in non-thermogenic conditions) and norepinephrine-mediated (occurring during thermogenic conditions) [107]. In rodent models, BAT is shown to be one of the most insulin-responsive tissues with respect to glucose-uptake [108] and is mediated via GLUT4, similar to that in WAT [109].

Animal studies suggest that chronic insulin deficiency reduces the thermogenic capacity of BAT [110],[111]. Furthermore, in type 1 diabetes mellitus glucose homeostasis is reverted to normalcy by increasing BAT quantity [112]. Contrarily, compensatory hyperinsulinaemia induces apoptosis of endothelial cells in rat BAT, thereby reducing BAT quantity [113]. This may explain reduced BAT activity observed in insulin-resistant states such as human obesity and T2DM in human PET-case series [16],[102]. In human PET studies, insulin-mediated glucose-uptake by BAT increased 5-fold (independent of perfusion) in comparison to WAT, and gene expression of *GLUT4* (Glucose transporter type 4) was higher in BAT than WAT [33]. In summary, it appears that insulin is required in maintenance of BAT thermogenic capacity, but the potential therapeutic role of insulin and insulin-related molecules in BAT manipulation is yet to be determined.

Central or peripheral intravenous leptin administration in rats is shown to increase insulin stimulated glucose utilisation, and to favour expression of uncoupling proteins predominantly through central pathways of increasing sympathetic tone [114],[115]. However, the lack of success of human recombinant leptin infusions on weight loss in obese subjects [116], and adverse cardiovascular profile of hypertension, left ventricular dysfunction, and possible cardiovascular risk [117] may need to be factored in for contemplating leptin route of BAT activation. Adiponectin is noted to inhibit UCP-1 gene expression by suppression of β3-adrenergic receptor in rats [118]. Conversely, adiponectin levels were significantly higher in BAT compared to WAT in active phaeochromocytoma patients, and consequently serum adiponectin levels markedly reduced following adrenalectomy [119]. The relation between BAT and adiponectin in humans is yet to be clarified before considering on therapeutic prospects.

### Endocannabinoids and BAT

Acting centrally and peripherally, the endocannabinoid system positively regulates appetite and energy balance [120] and has a role in adipose tissue metabolism [121], mainly through cannabinoid receptors (CB1 and CB2), and their natural endogenous ligands anandamide and 2-arachidonoyl glycerol [120]. In rodents, weight-loss associated with chronic CB1 antagonism was accompanied by increased energy expenditure, enhanced insulin-stimulated glucose utilisation, and marked activation of BAT thermogenesis [122]. Similar mice studies have shown a sustained increase of BAT temperature and up-regulation of UCP1 on CB1 blockade [123]. Through peripheral CB1 receptor inhibition, *in vitro* murine white adipocytes trans-differentiate into a mitochondria rich, thermogenic BAT phenotype [124]. Experiments with BAT denervation have attenuated such browning responses, indicating that central regulation is essential. Recent withdrawal of rimonabant from the market owing to concerns regarding an adverse psychotropic profile, poses a problem for CB1 being a target for activation of brown fat, unless a more selective peripheral blocker of CB1 is identified. Table 2 enlists effect of various hormones on BAT and possible therapeutic options through manipulation of individual hormonal actions.

**Table 2:** **Effect of hormones on BAT and possible therapeutic options**

Hormone	Influence on BAT	Probable BAT therapeutic suggestions
Epinephrine	+ve	Selective human β3 receptor agonists
T3	+ve	TR β selective agonists- GC-40, KB-41
Testosterone	-ve	To be determined
Estradiol	+/− (? dual effect)	Selective estrogen receptor modulators (SERM)
Progesterone	+ve	To be determined
DHEA	+ve	To be determined
IGF-1	Probably + ve	Recombinant human IGF-1 or truncated IGF-1
GH	+ve at higher dose	To be determined
Insulin	Unclear	To be determined
Cortisol	-ve	To be determined
Prolactin	-ve	Bromocriptine, pure prolactin receptor antagonists eg., ∆1–9-G129R- hPrl (∆1–9)
Aldosterone	-ve	Eplerenone, Spironolactone
Endocannabinoids	-ve	Peripheral CB1 antagonists

### Current trends in BAT therapeutics

Given that adult humans have BAT, it is important to explore BAT manipulation as a means of promoting weight-loss through enhanced energy expenditure via BAT manipulation. In addition to augmentation of BAT content and/or enhancement of BAT activity, other approaches include trans-differentiation of non-BAT progenitors into BAT pre-adipocytes, and surgical implantation of BAT. Development of novel BAT-related therapies will require a complete understanding of the embryological and transcriptional mechanisms of BAT specification and development in human models. We also need to characterize and confirm the physical and genetic attributes of BAT including anatomical and histological distributions of human BAT. Further challenges will be to develop a sustained long-term BAT stimulating or recruiting molecular circuit with adequate knowledge of counter-regulatory mechanisms for an acceptable safety profile, and to identify a reliable and safe imaging modality to monitor the effects of such therapies on BAT once developed and administered.

Several transcriptional regulators of brown adipocyte differentiation are described in rodents, with some revealing promising effects even in human models. Irisin is a 112-amino-acid polypeptide hormone, and is a cleaved and secreted fragment of fibronectin type III domain containing 5 (FNDC5) membrane protein, in turn released by muscle through increased PGC-1α expression following exercise in both rodents and humans [125]. Irisin showed a powerful browning effect on certain white adipose tissues in mice, both in culture and *in vivo*[125]. Human irisin is believed to be identical to mouse irisin, and in healthy adult subjects showed a 2-fold increase in plasma levels following 10 weeks of supervised endurance exercise training, as compared to the non-exercised state [125]. This PGC-1α dependent myokine alludes to the super-added beneficial effects of exercise via BAT, which need to be further explored.

The PRDM16-C/EBP-β transcriptional complex acts in Myogenic Factor-5 (Myf5) positive myoblastic precursors or pre-adipocytes to drive the thermogenic program with co-activation of PPAR-γ and PGC-1α [126],[127]. The cAMP-dependent thermogenic program is potentiated by Forkhead Box Protein C2 (FOXC2) [128] and PRDM16 and repressed by receptor-interacting protein-140 (RIP140) [129] (Figure 1). Other transcriptional regulators of Bone Morphogenic Protein-7 (BMP7) [130], Fibroblast Derived Growth Factor-21 (FGF21) [131], PPAR-γ ligands [132] and Atrial Natriuretic Peptide (ANP) [133], have been described in rodents. The transcripted cells through these various regulators are termed as BeAT as opposed to classical BAT and the success of these compounds depend upon extrapolating the gains in human models.

The discovery of brown adipocyte stem/progenitor cells, CD34+ in skeletal muscle [134] and human multi-potent adipose derived stem cells (hMADs) in subcutaneous tissue [135] in adult humans, serve as novel molecular targets for the development of BAT therapeutics as they have self-renewing capacity, and hence are expandable. In response to specific agents, muscle-derived CD34+ cells differentiate exclusively into brown adipocytes [134]. The WAT-derived hMADs, in contrast, first differentiate into WAT and following chronic exposure to PPAR-γ co-activators, gain brown adipose phenotype [135]. These human cell models provide a unique opportunity to study the formation and energy dissipation functions of human brown adipocytes, whilst simultaneously exploring therapeutic options. Such cells can potentially be externally induced into BAT, expanded and implanted back as an autologous implantation for metabolic beneficial effects as shown in recent mouse models [136]. Subcutaneous transplantation of embryonic BAT corrected type 1 diabetes in immune-competent mice as evidenced by reversal of diabetes symptoms, weight regain and normalization of glucose tolerance and the mice that remained euglycaemic 6-months following the procedure [137].

## Conclusion

There is compelling evidence to suggest that targeting cellular bioenergetics will yield an effective anti-obesity therapy. There are also complex practical concerns to be addressed. Recent key advances in the fields of molecular cell biology and metabolic science have raised relevant questions relating to the duration of the acquired BAT-like properties of cells following transcriptional regulation, the long-term fate of transcriptionally converted non-BAT (BeAT) tissues, the total amount of inactive BAT in humans and the fate of inter-scapular BAT in infants. Compensatory enhancement of appetite through central feedback regulation via complex neurological circuits following sustained chronic peripheral energy loss is a concern. Therefore, combining novel therapies that enhance BAT activity with an appetite-suppressant may be required. Therapeutic manipulation of peripheral energy expenditure through increasing BAT quantity and/or activity remains one of the most promising strategies for the successful prevention and management of human obesity. Although there are significant hurdles, there is also great potential for BAT manipulation to promote weight-loss through enhanced facultative metabolism.

## Authors’ information

NLR is a Clinical Lecturer in Division of Metabolic and Vascular Health (DMVH), University of Warwick, and Honorary Consultant in Diabetes & Endocrinology, University Hospitals Coventry and Warwickshire NHS Trust (UHCW). BKT is an Associate Clinical Professor in Division of Reproductive Health, University of Warwick and Consultant in Obstetrics and Gynaecology, UHCW. TMB is an Associate Professor in DMVH, University of Warwick and Consultant in Diabetes & Endocrinology, UHCW. HSR is a Senior Lecturer in DMVH, University of Warwick and Clinical Director/Consultant in Diabetes & Endocrinology, UHCW.

## Authors’ original submitted files for images

Below are the links to the authors’ original submitted files for images. Authors’ original file for figure 1

## Abbreviations

BAT: Brown adipose tissue
WHO: World Health Organization
T2DM: Type 2 diabetes mellitus
FDG: 18-Fluoro-labelled- 2-deoxyglucose
PET-CT: Positron Emission Tomography- Computed Tomography
WAT: White adipose tissue
BeAT: Beige adipose tissue
UCP-1: Uncoupling protein- 1
ATP: Adenosine tri-phosphate
AEE: Activity energy expenditure
TEE: Total energy expenditure
RMR: Resting metabolic rate
NST: Non-shivering thermogenesis
DIT: Diet-induced thermogenesis
NEFA: Non-esterified fatty acids
TH: Thyroid hormone
TR: Thyroid receptor
D2: type-2-deiodinase
T4: Thyroxine
T3: Thyronine
c-AMP: cyclic adenosine-mono-phosphate
PKA: Protein kinase-A
Lpl: Lipoprotein lipase
PPAR-γ: Peroxisome proliferator receptor activated-gamma
aP2: Adipocyte-specific fatty acid binding protein
IL-6: Interleukin-6
TNF-α: Tumour necrosis factor-alpha
MCP-1: Monocyte chemoattractant protein
GH: Growth Hormone
IGF-1: Insulin Growth Factor-1
C/EBP- α: CCAAT-enhancer binding protein- alpha
PRDM-16: PR domain containing 16
PGC-1α: Peroxisome proliferator activated receptor gamma coactivator 1- alpha
CB-1: Cannabinoid receptor-1
ER-α: Oestrogen receptor-alpha
E2: 17-β-Oestradiol
NRF-1: Nuclear respiratory transcriptional factor-1
GABPA: GA-binding protein transcription protein- alpha
TFAM: Mitochondrial transcription factor-A
PTEN: Phosphatase and tensin homolog deleted on chromosome 10
AR: Adrenergic receptor
SERMs: Selective oestrogen receptor modulators
GLUT-4: Glucose transporter type 4
FNDC-5: Fibronectin type 3 domain containing 5
Myf-5: Myogenic factor-5
FOXC2: Forkhead box protein C2
RIP140: Receptor interacting protein-140
BMP-7: Bone morphogenic protein-7
FGF21: Fibroblast derived growth factor-21
hMADs: Human multi-potent adipocyte derived stem cells

## References

[1] WHO global infobase: data on overweight and obesity.., [http://www.who.int/mediacentre/factsheets/fs311/en/]

[2] Larsson B, Svardsudd K, Welin L, Wilhelmsen L, Bjorntorp P, Tibblin G (1984). Abdominal adipose tissue distribution, obesity, and risk of cardiovascular disease and death: 13 year follow up of participants in the study of men born in 1913. Br Med J (Clin Res Ed).

[3] Lim SS, Vos T, Flaxman AD, Danaei G, Shibuya K, Adair-Rohani H, Amann M, Anderson HR, Andrews KG, Aryee M, Lim SS, Vos T, Flaxman AD, Danaei G, Shibuya K, Adair-Rohani H, Amann M, Anderson HR, Andrews KG, Aryee M, Atkinson C, Bacchus LJ, Bahalim AN, Balakrishnan K, Balmes J, Barker-Collo S, Baxter A, Bell ML, Blore JD, Blyth F (2012). A comparative risk assessment of burden of disease and injury attributable to 67 risk factors and risk factor clusters in 21 regions, 1990–2010: a systematic analysis for the Global Burden of Disease Study 2010. Lancet.

[4] Ng M, Fleming T, Robinson M, Thomson B, Graetz N, Margono C, Mullany EC, Biryukov S, Abbafati C, Abera SF, Abraham JP, Abu-Rmeileh NM, Achoki T, AlBuhairan FS, Alemu ZA, Alfonso R, Ali MK, Ali R, Guzman NA, Ammar W, Anwari P, Banerjee A, Barquera S, Basu S, Bennett DA, Bhutta Z, Blore J, Cabral N, Nonato IC, Chang JC: Global, regional, and national prevalence of overweight and obesity in children and adults during 1980–2013: a systematic analysis for the Global Burden of Disease Study 2013.*Lancet* 2014. e-publication ahead of print., 10.1016/S0140-6736(14)60460-8 PMC4624264 24880830

[5] Scott A, Ejikeme CS, Clottey EN, Thomas JG (2013). Obesity in sub-Saharan Africa: development of an ecological theoretical framework. Health Promot Int.

[6] Sumithran P, Prendergast LA, Delbridge E, Purcell K, Shulkes A, Kriketos A, Proietto J (2011). Long-term persistence of hormonal adaptations to weight loss. N Engl J Med.

[7] Rosenbaum M, Hirsch J, Gallagher DA, Leibel RL (2008). Long-term persistence of adaptive thermogenesis in subjects who have maintained a reduced body weight. Am J Clin Nutr.

[8] Padwal R, Li SK, Lau DC (2004). Long-term pharmacotherapy for obesity and overweight. Cochrane Database Syst Rev.

[9] Dixon JB, Zimmet P, Alberti KG, Rubino F (2011). Bariatric surgery: an IDF statement for obese Type 2 diabetes. Surg Obes Relat Dis.

[10] Padwal RS, Majumdar SR (2007). Drug treatments for obesity: orlistat, sibutramine, and rimonabant. Lancet.

[11] Tseng YH, Cypess AM, Kahn CR (2010). Cellular bioenergetics as a target for obesity therapy. Nat Rev Drug Discov.

[12] Wells JC (2009). Thrift: a guide to thrifty genes, thrifty phenotypes and thrifty norms. Int J Obes (Lond).

[13] Fawcett DW, Jones IC (1949). The effects of hypophysectomy, adrenalectomy and of thiouracil feeding on the cytology of brown adipose tissue. Endocrinology.

[14] Virtanen KA, Lidell ME, Orava J, Heglind M, Westergren R, Niemi T, Taittonen M, Laine J, Savisto NJ, Enerback S, Nuutila P (2009). Functional brown adipose tissue in healthy adults. N Engl J Med.

[15] Hany TF, Gharehpapagh E, Kamel EM, Buck A, Himms-Hagen J, von Schulthess GK (2002). Brown adipose tissue: a factor to consider in symmetrical tracer uptake in the neck and upper chest region. Eur J Nucl Med Mol Imaging.

[16] Cypess AM, Lehman S, Williams G, Tal I, Rodman D, Goldfine AB, Kuo FC, Palmer EL, Tseng YH, Doria A, Kolodny GM, Kahn CR (2009). Identification and importance of brown adipose tissue in adult humans. N Engl J Med.

[17] Guerra C, Koza RA, Yamashita H, Walsh K, Kozak LP (1998). Emergence of brown adipocytes in white fat in mice is under genetic control: effects on body weight and adiposity. J Clin Invest.

[18] Lean ME, James WP, Jennings G, Trayhurn P (1986). Brown adipose tissue in patients with phaeochromocytoma. Int J Obes (Lond).

[19] Lahesmaa M, Orava J, Schalin-Jantti C, Soinio M, Hannukainen JC, Noponen T, Kirjavainen A, Iida H, Kudomi N, Enerback S, Virtanen KA, Nuutila P (2014). Hyperthyroidism increases brown fat metabolism in humans. J Clin Endocrinol Metab.

[20] Enerback S (2010). Brown adipose tissue in humans. Int J Obes (Lond).

[21] Wu J, Bostrom P, Sparks LM, Ye L, Choi JH, Giang AH, Khandekar M, Virtanen KA, Nuutila P, Schaart G, Huang K, Tu H, Van Marken Lichtenbelt WD, Hoeks J, Enerbäck S, Schrauwen P, Spiegelman BM (2012). Beige adipocytes are a distinct type of thermogenic fat cell in mouse and human. Cell.

[22] Whittle AJ, Lopez M, Vidal-Puig A (2011). Using brown adipose tissue to treat obesity - the central issue. Trends Mol Med.

[23] van Marken Lichtenbelt WD, Schrauwen P (2011). Implications of nonshivering thermogenesis for energy balance regulation in humans. Am J Physiol Regul Integr Comp Physiol.

[24] Westerterp KR, Wilson SA, Rolland V (1999). Diet induced thermogenesis measured over 24 h in a respiration chamber: effect of diet composition. Int J Obes Relat Metab Disord.

[25] Saito M, Okamatsu-Ogura Y, Matsushita M, Watanabe K, Yoneshiro T, Nio-Kobayashi J, Iwanaga T, Miyagawa M, Kameya T, Nakada K, Kawai Y, Tsujisaki M (2009). High incidence of metabolically active brown adipose tissue in healthy adult humans: effects of cold exposure and adiposity. Diabetes.

[26] van Marken Lichtenbelt WD, Vanhommerig JW, Smulders NM, Drossaerts JM, Kemerink GJ, Bouvy ND, Schrauwen P, Teule GJ (2009). Cold-activated brown adipose tissue in healthy men. N Engl J Med.

[27] Nicholls DG, Locke RM (1984). Thermogenic mechanisms in brown fat. Physiol Rev.

[28] Kozak LP (2010). Brown fat and the myth of diet-induced thermogenesis. Cell Metab.

[29] Ouellet V, Labbe SM, Blondin DP, Phoenix S, Guerin B, Haman F, Turcotte EE, Richard D, Carpentier AC (2012). Brown adipose tissue oxidative metabolism contributes to energy expenditure during acute cold exposure in humans. J Clin Invest.

[30] Rothwell NJ, Stock MJ (1983). Luxuskonsumption, diet-induced thermogenesis and brown fat: the case in favour. Clin Sci (Lond).

[31] Yoneshiro T, Aita S, Matsushita M, Kameya T, Nakada K, Kawai Y, Saito M (2011). Brown adipose tissue, whole-body energy expenditure, and thermogenesis in healthy adult men. Obesity (Silver Spring).

[32] Muzik O, Mangner TJ, Granneman JG (2012). Assessment of oxidative metabolism in brown fat using PET imaging. Front Endocrinol (Lausanne).

[33] Orava J, Nuutila P, Lidell ME, Oikonen V, Noponen T, Viljanen T, Scheinin M, Taittonen M, Niemi T, Enerback S, Virtanen KA (2011). Different metabolic responses of human brown adipose tissue to activation by cold and insulin. Cell Metab.

[34] Bartelt A, Bruns OT, Reimer R, Hohenberg H, Ittrich H, Peldschus K, Kaul MG, Tromsdorf UI, Weller H, Waurisch C, Eychmüller A, Gordts PL, Rinninger F, Bruegelmann K, Freund B, Nielsen P, Merkel M, Heeren J (2011). Brown adipose tissue activity controls triglyceride clearance. Nat Med.

[35] Klitgaard HM, Dirks HB, Garlick WR, Barker SB (1952). Protein-bound iodine in various tissues after injection of elemental iodine. Endocrinology.

[36] Klieverik LP, Coomans CP, Endert E, Sauerwein HP, Havekes LM, Voshol PJ, Rensen PC, Romijn JA, Kalsbeek A, Fliers E (2009). Thyroid hormone effects on whole-body energy homeostasis and tissue-specific fatty acid uptake in vivo. Endocrinology.

[37] Silva JE (2006). Thermogenic mechanisms and their hormonal regulation. Physiol Rev.

[38] Jiang W, Miyamoto T, Kakizawa T, Sakuma T, Nishio S, Takeda T, Suzuki S, Hashizume K (2004). Expression of thyroid hormone receptor alpha in 3 T3-L1 adipocytes; triiodothyronine increases the expression of lipogenic enzyme and triglyceride accumulation. J Endocrinol.

[39] Viguerie N, Millet L, Avizou S, Vidal H, Larrouy D, Langin D (2002). Regulation of human adipocyte gene expression by thyroid hormone. J Clin Endocrinol Metab.

[40] Sheehan TE, Kumar PA, Hood DA (2004). Tissue-specific regulation of cytochrome c oxidase subunit expression by thyroid hormone. Am J Physiol Endocrinol Metab.

[41] Lopez M, Varela L, Vazquez MJ, Rodriguez-Cuenca S, Gonzalez CR, Velagapudi VR, Morgan DA, Schoenmakers E, Agassandian K, Lage R, Martínez de Morentin PB, Tovar S, Nogueiras R, Carling D, Lelliott C, Gallego R, Oresic M, Chatterjee K, Saha AK, Rahmouni K, Diéguez C, Vidal-Puig A (2010). Hypothalamic AMPK and fatty acid metabolism mediate thyroid regulation of energy balance. Nat Med.

[42] Brent GA (1994). The molecular basis of thyroid hormone action. N Engl J Med.

[43] Ribeiro MO, Carvalho SD, Schultz JJ, Chiellini G, Scanlan TS, Bianco AC, Brent GA (2001). Thyroid hormone–sympathetic interaction and adaptive thermogenesis are thyroid hormone receptor isoform–specific. J Clin Invest.

[44] Bianco AC, Silva JE (1988). Cold exposure rapidly induces virtual saturation of brown adipose tissue nuclear T3 receptors. Am J Physiol.

[45] Bryzgalova G, Effendic S, Khan A, Rehnmark S, Barbounis P, Boulet J, Dong G, Singh R, Shapses S, Malm J, Webb P, Baxter JD, Grover GJ (2008). Anti-obesity, anti-diabetic, and lipid lowering effects of the thyroid receptor beta subtype selective agonist KB-141. J Steroid Biochem Mol Biol.

[46] Grover GJ, Egan DM, Sleph PG, Beehler BC, Chiellini G, Nguyen NH, Baxter JD, Scanlan TS (2004). Effects of the thyroid hormone receptor agonist GC-1 on metabolic rate and cholesterol in rats and primates: selective actions relative to 3,5,3'-triiodo-L-thyronine. Endocrinology.

[47] Amorim BS, Ueta CB, Freitas BC, Nassif RJ, Gouveia CH, Christoffolete MA, Moriscot AS, Lancelloti CL, Llimona F, Barbeiro HV, de Souza HP, Catanozi S, Passarelli M, Aoki MS, Bianco AC, Ribeiro MO (2009). A TRbeta-selective agonist confers resistance to diet-induced obesity. J Endocrinol.

[48] Lee JY, Takahashi N, Yasubuchi M, Kim YI, Hashizaki H, Kim MJ, Sakamoto T, Goto T, Kawada T (2011). Triiodothyronine induces UCP1 expression and mitochondrial biogenesis in human adipocytes. Am J Physiol Cell Physiol.

[49] Skarulis MC, Celi FS, Mueller E, Zemskova M, Malek R, Hugendubler L, Cochran C, Solomon J, Chen C, Gorden P (2010). Thyroid hormone induced brown adipose tissue and amelioration of diabetes in a patient with extreme insulin resistance. J Clin Endocrinol Metab.

[50] Simonsen L, Bulow J, Madsen J, Christensen NJ (1992). Thermogenic response to epinephrine in the forearm and abdominal subcutaneous adipose tissue. Am J Physiol.

[51] Simonsen L, Stallknecht B, Bulow J (1993). Contribution of skeletal muscle and adipose tissue to adrenaline-induced thermogenesis in man. Int J Obes Relat Metab Disord.

[52] Ricquier D, Nechad M, Mory G (1982). Ultrastructural and biochemical characterization of human brown adipose tissue in pheochromocytoma. J Clin Endocrinol Metab.

[53] English JT, Patel SK, Flanagan MJ (1973). Association of pheochromocytomas with brown fat tumors. Radiology.

[54] Melicow MM (1957). Hibernating fat and pheochromocytoma. AMA Arch Pathol.

[55] Bouillaud F, Ricquier D, Mory G, Thibault J (1984). Increased level of mRNA for the uncoupling protein in brown adipose tissue of rats during thermogenesis induced by cold exposure or norepinephrine infusion. J Biol Chem.

[56] Hadi M, Chen CC, Whatley M, Pacak K, Carrasquillo JA (2007). Brown fat imaging with (18)F-6-fluorodopamine PET/CT, (18)F-FDG PET/CT, and (123)I-MIBG SPECT: a study of patients being evaluated for pheochromocytoma. J Nucl Med.

[57] Wang Q, Zhang M, Ning G, Gu W, Su T, Xu M, Li B, Wang W (2011). Brown adipose tissue in humans is activated by elevated plasma catecholamines levels and is inversely related to central obesity. PLoS One.

[58] Himms-Hagen J, Cui J, Danforth E, Taatjes DJ, Lang SS, Waters BL, Claus TH (1994). Effect of CL-316,243, a thermogenic beta 3-agonist, on energy balance and brown and white adipose tissues in rats. Am J Physiol.

[59] Susulic VS, Frederich RC, Lawitts J, Tozzo E, Kahn BB, Harper ME, Himms-Hagen J, Flier JS, Lowell BB (1995). Targeted disruption of the beta 3-adrenergic receptor gene. J Biol Chem.

[60] Arch JR (2008). The discovery of drugs for obesity, the metabolic effects of leptin and variable receptor pharmacology: perspectives from beta3-adrenoceptor agonists. Naunyn Schmiedebergs Arch Pharmacol.

[61] Larsen TM, Toubro S, van Baak MA, Gottesdiener KM, Larson P, Saris WH, Astrup A (2002). Effect of a 28-d treatment with L-796568, a novel beta(3)-adrenergic receptor agonist, on energy expenditure and body composition in obese men. Am J Clin Nutr.

[62] van Baak MA, Hul GB, Toubro S, Astrup A, Gottesdiener KM, DeSmet M, Saris WH (2002). Acute effect of L-796568, a novel beta 3-adrenergic receptor agonist, on energy expenditure in obese men. Clin Pharmacol Ther.

[63] Joshi PV, Lele VR (2012). Unexpected visitor on FDG PET/CT–brown adipose tissue (BAT) in mesentery in a case of retroperitoneal extra-adrenal pheochromocytoma: is the BAT activation secondary to catecholamine-secreting pheochromocytoma?. Clin Nucl Med.

[64] Cheng W, Zhu Z, Jin X, Chen L, Zhuang H, Li F (2012). Intense FDG activity in the brown adipose tissue in omental and mesenteric regions in a patient with malignant pheochromocytoma. Clin Nucl Med.

[65] Feldman D (1978). Evidence that brown adipose tissue is a glucocorticoid target organ. Endocrinology.

[66] Strack AM, Sebastian RJ, Schwartz MW, Dallman MF (1995). Glucocorticoids and insulin: reciprocal signals for energy balance. Am J Physiol.

[67] Soumano K, Desbiens S, Rabelo R, Bakopanos E, Camirand A, Silva JE (2000). Glucocorticoids inhibit the transcriptional response of the uncoupling protein-1 gene to adrenergic stimulation in a brown adipose cell line. Mol Cell Endocrinol.

[68] Garrel DR (1997). Glucocorticoids and energy expenditure: relevance to the regulation of energy balance in man. Nutrition.

[69] Strack AM, Bradbury MJ, Dallman MF (1995). Corticosterone decreases nonshivering thermogenesis and increases lipid storage in brown adipose tissue. Am J Physiol.

[70] Feve B, Baude B, Krief S, Strosberg AD, Pairault J, Emorine LJ (1992). Inhibition by dexamethasone of beta 3-adrenergic receptor responsiveness in 3 T3-F442A adipocytes. Evidence for a transcriptional mechanism. J Biol Chem.

[71] Kiely J, Hadcock JR, Bahouth SW, Malbon CC (1994). Glucocorticoids down-regulate beta 1-adrenergic-receptor expression by suppressing transcription of the receptor gene. Biochem J.

[72] Vander Tuig JG, Ohshima K, Yoshida T, Romsos DR, Bray GA (1984). Adrenalectomy increases norepinephrine turnover in brown adipose tissue of obese (ob/ob) mice. Life Sci.

[73] Berthiaume M, Sell H, Lalonde J, Gelinas Y, Tchernof A, Richard D, Deshaies Y (2004). Am J Physiol Regul Integr Comp Physiol. Am J Physiol Regul Integr Comp Physiol.

[74] Ashizawa N, Takagi M, Seto S, Suzuki S, Yano K (2007). Serum adiponectin and leptin in a patient with Cushing's syndrome before and after adrenalectomy. Intern Med.

[75] Zennaro MC, Le Menuet D, Viengchareun S, Walker F, Ricquier D, Lombes M (1998). Hibernoma development in transgenic mice identifies brown adipose tissue as a novel target of aldosterone action. J Clin Invest.

[76] Penfornis P, Viengchareun S, Le Menuet D, Cluzeaud F, Zennaro MC, Lombes M (2000). The mineralocorticoid receptor mediates aldosterone-induced differentiation of T37i cells into brown adipocytes. Am J Physiol Endocrinol Metab.

[77] Viengchareun S, Penfornis P, Zennaro MC, Lombes M (2001). Mineralocorticoid and glucocorticoid receptors inhibit UCP expression and function in brown adipocytes. Am J Physiol Endocrinol Metab.

[78] Kraus D, Jager J, Meier B, Fasshauer M, Klein J (2005). Aldosterone inhibits uncoupling protein-1, induces insulin resistance, and stimulates proinflammatory adipokines in adipocytes. Horm Metab Res.

[79] Hoppmann J, Perwitz N, Meier B, Fasshauer M, Hadaschik D, Lehnert H, Klein J (2010). The balance between gluco- and mineralo-corticoid action critically determines inflammatory adipocyte responses. J Endocrinol.

[80] Marzolla V, Armani A, Zennaro MC, Cinti F, Mammi C, Fabbri A, Rosano GM, Caprio M (2012). The role of the mineralocorticoid receptor in adipocyte biology and fat metabolism. Mol Cell Endocrinol.

[81] Feraco A, Armani A, Mammi C, Fabbri A, Rosano GM, Caprio M (2013). Role of mineralocorticoid receptor and renin-angiotensin-aldosterone system in adipocyte dysfunction and obesity. J Steroid Biochem Mol Biol.

[82] Al-Shoumer KA, Page B, Thomas E, Murphy M, Beshyah SA, Johnston DG (1996). Effects of four years' treatment with biosynthetic human growth hormone (GH) on body composition in GH-deficient hypopituitary adults. Eur J Endocrinol.

[83] Hoffman AR, Kuntze JE, Baptista J, Baum HB, Baumann GP, Biller BM, Clark RV, Cook D, Inzucchi SE, Kleinberg D, Klibanski A, Phillips LS, Ridgway EC, Robbins RJ, Schlechte J, Sharma M, Thorner MO, Vance ML (2004). Growth hormone (GH) replacement therapy in adult-onset gh deficiency: effects on body composition in men and women in a double-blind, randomized, placebo-controlled trial. J Clin Endocrinol Metab.

[84] Hioki C, Yoshida T, Kogure A, Takakura Y, Umekawa T, Yoshioka K, Shimatsu A, Yoshikawa T (2004). Effects of growth hormone (GH) on mRNA levels of uncoupling proteins 1, 2, and 3 in brown and white adipose tissues and skeletal muscle in obese mice. Horm Metab Res.

[85] Lorenzo M, Valverde AM, Teruel T, Benito M (1993). IGF-I is a mitogen involved in differentiation-related gene expression in fetal rat brown adipocytes. J Cell Biol.

[86] Valverde AM, Benito M, Lorenzo M (1991). Proliferation of fetal brown adipocyte primary cultures: relationship with the genetic expression of glucose 6 phosphate dehydrogenase. Exp Cell Res.

[87] Porras A, Alvarez AM, Valladares A, Benito M (1997). TNF-alpha induces apoptosis in rat fetal brown adipocytes in primary culture. FEBS Lett.

[88] Guerra C, Benito M, Fernandez M (1994). IGF-I induces the uncoupling protein gene expression in fetal rat brown adipocyte primary cultures: role of C/EBP transcription factors. Biochem Biophys Res Commun.

[89] Duchamp C, Burton KA, Geloen A, Dauncey MJ (1997). Transient upregulation of IGF-I gene expression in brown adipose tissue of cold-exposed rats. Am J Physiol.

[90] Symonds ME, Mostyn A, Pearce S, Budge H, Stephenson T (2003). Endocrine and nutritional regulation of fetal adipose tissue development. J Endocrinol.

[91] Viengchareun S, Servel N, Feve B, Freemark M, Lombes M, Binart N (2008). Prolactin receptor signaling is essential for perinatal brown adipocyte function: a role for insulin-like growth factor-2. PLoS One.

[92] Ben-Jonathan N, LaPensee CR, LaPensee EW (2008). What can we learn from rodents about prolactin in humans?. Endocr Rev.

[93] Pijl H, Ohashi S, Matsuda M, Miyazaki Y, Mahankali A, Kumar V, Pipek R, Iozzo P, Lancaster JL, Cincotta AH, DeFronzo RA (2000). Bromocriptine: a novel approach to the treatment of type 2 diabetes. Diabetes Care.

[94] Krol E, Martin SA, Huhtaniemi IT, Douglas A, Speakman JR (2011). Negative correlation between milk production and brown adipose tissue gene expression in lactating mice. J Exp Biol.

[95] Julien Auffret SV, Adeline M, Bruno F, Marc L, Nadine B (2011). Mice lacking prolactin receptor resist high-fat diet–induced obesity by browning of adipose tissue. Endocr Rev.

[96] Rodriguez-Cuenca S, Monjo M, Frontera M, Gianotti M, Proenza AM, Roca P (2007). Sex steroid receptor expression profile in brown adipose tissue: effects of hormonal status. Cell Physiol Biochem.

[97] Gaspard U (2009). Hyperinsulinaemia, a key factor of the metabolic syndrome in postmenopausal women. Maturitas.

[98] Nadal-Casellas A, Proenza AM, Llado I, Gianotti M (2011). Effects of ovariectomy and 17-beta estradiol replacement on rat brown adipose tissue mitochondrial function. Steroids.

[99] Rodriguez-Cuenca S, Monjo M, Gianotti M, Proenza AM, Roca P (2007). Expression of mitochondrial biogenesis-signaling factors in brown adipocytes is influenced specifically by 17beta-estradiol, testosterone, and progesterone. Am J Physiol Endocrinol Metab.

[100] Monjo M, Rodriguez AM, Palou A, Roca P (2003). Direct effects of testosterone, 17 beta-estradiol, and progesterone on adrenergic regulation in cultured brown adipocytes: potential mechanism for gender-dependent thermogenesis. Endocrinology.

[101] Rodriguez AM, Monjo M, Roca P, Palou A (2002). Opposite actions of testosterone and progesterone on UCP1 mRNA expression in cultured brown adipocytes. Cell Mol Life Sci.

[102] Ouellet V, Routhier-Labadie A, Bellemare W, Lakhal-Chaieb L, Turcotte E, Carpentier AC, Richard D (2011). Outdoor temperature, age, sex, body mass index, and diabetic status determine the prevalence, mass, and glucose-uptake activity of 18 F-FDG-detected BAT in humans. J Clin Endocrinol Metab.

[103] Ryu JW, Kim MS, Kim CH, Song KH, Park JY, Lee JD, Kim JB, Lee KU (2003). DHEA administration increases brown fat uncoupling protein 1 levels in obese OLETF rats. Biochem Biophys Res Commun.

[104] Gilsanz V, Hu HH, Kajimura S (2013). Relevance of brown adipose tissue in infancy and adolescence. Pediatr Res.

[105] Tseng YH, Kriauciunas KM, Kokkotou E, Kahn CR (2004). Differential roles of insulin receptor substrates in brown adipocyte differentiation. Mol Cell Biol.

[106] Tanti JF, Gremeaux T, Brandenburg D, Van Obberghen E, Le Marchand-Brustel Y (1986). Brown adipose tissue in lean and obese mice. Insulin-receptor binding and tyrosine kinase activity. Diabetes.

[107] Shimizu Y, Kielar D, Minokoshi Y, Shimazu T (1996). Noradrenaline increases glucose transport into brown adipocytes in culture by a mechanism different from that of insulin. Biochem J.

[108] Storlien LH, James DE, Burleigh KM, Chisholm DJ, Kraegen EW (1986). Fat feeding causes widespread in vivo insulin resistance, decreased energy expenditure, and obesity in rats. Am J Physiol.

[109] Shimizu Y, Nikami H, Tsukazaki K, Machado UF, Yano H, Seino Y, Saito M (1993). Increased expression of glucose transporter GLUT-4 in brown adipose tissue of fasted rats after cold exposure. Am J Physiol.

[110] Rothwell NJ, Stock MJ (1981). A role for insulin in the diet-induced thermogenesis of cafeteria-fed rats. Metabolism.

[111] Shibata H, Perusse F, Bukowiecki LJ (1987). The role of insulin in nonshivering thermogenesis. Can J Physiol Pharmacol.

[112] Gunawardana SC, Piston DW (2012). Reversal of type 1 diabetes in mice by brown adipose tissue transplant. Diabetes.

[113] Markelic M, Velickovic K, Golic I, Otasevic V, Stancic A, Jankovic A, Vucetic M, Buzadzic B, Korac B, Korac A (2011). Endothelial cell apoptosis in brown adipose tissue of rats induced by hyperinsulinaemia: the possible role of TNF-alpha. Eur J Histochem.

[114] Rouru J, Cusin I, Zakrzewska KE, Jeanrenaud B, Rohner-Jeanrenaud F (1999). Effects of intravenously infused leptin on insulin sensitivity and on the expression of uncoupling proteins in brown adipose tissue. Endocrinology.

[115] Enriori PJ, Sinnayah P, Simonds SE, Garcia Rudaz C, Cowley MA (2011). Leptin action in the dorsomedial hypothalamus increases sympathetic tone to brown adipose tissue in spite of systemic leptin resistance. J Neurosci.

[116] Heymsfield SB, Greenberg AS, Fujioka K, Dixon RM, Kushner R, Hunt T, Lubina JA, Patane J, Self B, Hunt P, McCamish M (1999). Recombinant leptin for weight loss in obese and lean adults: a randomized, controlled, dose-escalation trial. Jama.

[117] Koh KK, Park SM, Quon MJ (2008). Leptin and cardiovascular disease: response to therapeutic interventions. Circulation.

[118] Qiao L, Yoo H, Bosco C, Lee B, Feng GS, Schaack J, Chi NW, Shao J (2014). Adiponectin reduces thermogenesis by inhibiting brown adipose tissue activation in mice. Diabetologia.

[119] Iacobellis G, Di Gioia C, Petramala L, Chiappetta C, Serra V, Zinnamosca L, Marinelli C, Ciardi A, De Toma G, Letizia C (2013). Brown fat expresses adiponectin in humans. Int J Endocrinol.

[120] Rinaldi-Carmona M, Barth F, Heaulme M, Shire D, Calandra B, Congy C, Martinez S, Maruani J, Neliat G, Caput D, Ferrara P, Soubrié P, Brelière JC, Le Fur G (1994). SR141716A, a potent and selective antagonist of the brain cannabinoid receptor. FEBS Lett.

[121] Muccioli GG, Naslain D, Backhed F, Reigstad CS, Lambert DM, Delzenne NM, Cani PD (2010). The endocannabinoid system links gut microbiota to adipogenesis. Mol Syst Biol.

[122] Bajzer M, Olivieri M, Haas MK, Pfluger PT, Magrisso IJ, Foster MT, Tschop MH, Krawczewski-Carhuatanta KA, Cota D, Obici S (2011). Cannabinoid receptor 1 (CB1) antagonism enhances glucose utilisation and activates brown adipose tissue in diet-induced obese mice. Diabetologia.

[123] Verty AN, Allen AM, Oldfield BJ (2009). The effects of rimonabant on brown adipose tissue in rat: implications for energy expenditure. Obesity (Silver Spring).

[124] Perwitz N, Wenzel J, Wagner I, Buning J, Drenckhan M, Zarse K, Ristow M, Lilienthal W, Lehnert H, Klein J (2010). Cannabinoid type 1 receptor blockade induces transdifferentiation towards a brown fat phenotype in white adipocytes. Diabetes Obes Metab.

[125] Bostrom P, Wu J, Jedrychowski MP, Korde A, Ye L, Lo JC, Rasbach KA, Bostrom EA, Choi JH, Long JZ, Kajimura S, Zingaretti MC, Vind BF, Tu H, Cinti S, Højlund K, Gygi SP, Spiegelman BM (2012). A PGC1-alpha-dependent myokine that drives brown-fat-like development of white fat and thermogenesis. Nature.

[126] Seale P, Bjork B, Yang W, Kajimura S, Chin S, Kuang S, Scime A, Devarakonda S, Conroe HM, Erdjument-Bromage H, Tempst P, Rudnicki MA, Beier DR, Spiegelman BM (2008). PRDM16 controls a brown fat/skeletal muscle switch. Nature.

[127] Kajimura S, Seale P, Kubota K, Lunsford E, Frangioni JV, Gygi SP, Spiegelman BM (2009). Initiation of myoblast to brown fat switch by a PRDM16-C/EBP-beta transcriptional complex. Nature.

[128] Cederberg A, Gronning LM, Ahren B, Tasken K, Carlsson P, Enerback S (2001). FOXC2 is a winged helix gene that counteracts obesity, hypertriglyceridemia, and diet-induced insulin resistance. Cell.

[129] Hallberg M, Morganstein DL, Kiskinis E, Shah K, Kralli A, Dilworth SM, White R, Parker MG, Christian M (2008). A functional interaction between RIP140 and PGC-1alpha regulates the expression of the lipid droplet protein CIDEA. Mol Cell Biol.

[130] Tseng YH, Kokkotou E, Schulz TJ, Huang TL, Winnay JN, Taniguchi CM, Tran TT, Suzuki R, Espinoza DO, Yamamoto Y, Ahrens MJ, Dudley AT, Norris AW, Kulkarni RN, Kahn CR (2008). New role of bone morphogenetic protein 7 in brown adipogenesis and energy expenditure. Nature.

[131] Beenken A, Mohammadi M (2009). The FGF family: biology, pathophysiology and therapy. Nat Rev Drug Discov.

[132] Wilson-Fritch L, Nicoloro S, Chouinard M, Lazar MA, Chui PC, Leszyk J, Straubhaar J, Czech MP, Corvera S (2004). Mitochondrial remodeling in adipose tissue associated with obesity and treatment with rosiglitazone. J Clin Invest.

[133] Bordicchia M, Liu D, Amri EZ, Ailhaud G, Dessi-Fulgheri P, Zhang C, Takahashi N, Sarzani R, Collins S (2012). Cardiac natriuretic peptides act via p38 MAPK to induce the brown fat thermogenic program in mouse and human adipocytes. J Clin Invest.

[134] Crisan M, Casteilla L, Lehr L, Carmona M, Paoloni-Giacobino A, Yap S, Sun B, Leger B, Logar A, Penicaud L, Schrauwen P, Cameron-Smith D, Russell AP, Péault B, Giacobino JP (2008). A reservoir of brown adipocyte progenitors in human skeletal muscle. Stem Cells.

[135] Elabd C, Chiellini C, Carmona M, Galitzky J, Cochet O, Petersen R, Penicaud L, Kristiansen K, Bouloumie A, Casteilla L, Dani C, Ailhaud G, Amri EZ (2009). Human multipotent adipose-derived stem cells differentiate into functional brown adipocytes. Stem Cells.

[136] Stanford-RJ-WM KI, Ding AN, Kristy T, Hitchcox KM, Dae Young J, Yong Jin L, Kim JK, Hirshman MF, Yu-Hua T, Goodyear LJ (2011). Transplantation of Brown Adipose Tissue Exerts Beneficial Effects on Glucose Homeostasis.

[137] David W, Piston SCG (2011). Reversal of Type 1 Diabetes by Brown Adipose Tissue Transplant.

