# Supplementary figures and images for: Brown adipose tissue: endocrine determinants of function and therapeutic manipulation as a novel treatment strategy for obesity
Source: BMC Obes. 2014 Aug 22;1:13. doi: 10.1186/s40608-014-0013-5 (PMC4765227; doi:10.1186/s40608-014-0013-5)

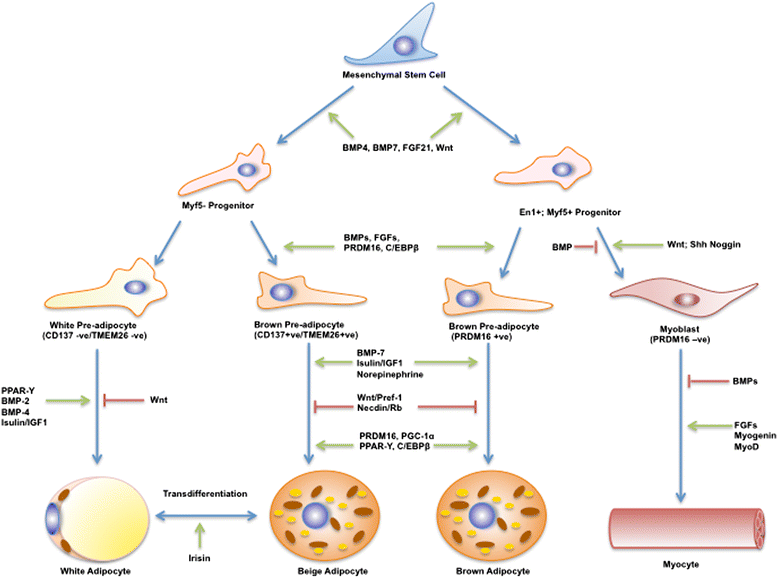

Supplement: Supplementary file 1 — Authors’ original file for figure 1 [file 40608_2014_13_MOESM1_ESM.gif]
